# The importance of metagenomic surveys to microbial ecology: or why Darwin would have been a metagenomic scientist

**DOI:** 10.1186/2042-5783-1-5

**Published:** 2011-06-14

**Authors:** Jack A Gilbert, Ronald O'Dor, Nicholas King, Timothy M Vogel

**Affiliations:** 1 Argonne National Laboratory, 9700 South Cass Avenue, Argonne, IL 60439, USA; 2 Department of Ecology and Evolution, University of Chicago, 5640 South Ellis Avenue, Chicago, IL 60637, USA; 3 Department of Biology, Life Science Centre, Dalhousie University, 1355 Oxford Street, Halifax, NS, B3H 4J1, Canada; 4 Global Biodiversity Information Facility, Universitetsarken 15, DK-2100 Copenhagen, Denmark; 5 Environmental Microbial Genomics, Ecole Centrale de Lyon, Université de Lyon, 36 avenue Guy de Collongue, 69134 Ecully, France

## Abstract

Scientific discovery is incremental. The Merriam-Webster definition of 'Scientific Method' is "principles and procedures for the systematic pursuit of knowledge involving the recognition and formulation of a problem, the collection of data through observation and experiment, and the formulation and testing of hypotheses". Scientists are taught to be excellent observers, as observations create questions, which in turn generate hypotheses. After centuries of science we tend to assume that we have enough observations to drive science, and enable the small steps and giant leaps which lead to theories and subsequent testable hypotheses. One excellent example of this is Charles Darwin's Voyage of the Beagle, which was essentially an opportunistic survey of biodiversity. Today, obtaining funding for even small-scale surveys of life on Earth is difficult; but few argue the importance of the theory that was generated by Darwin from his observations made during this epic journey. However, these observations, even combined with the parallel work of Alfred Russell Wallace at around the same time have still not generated an indisputable 'law of biology'. The fact that evolution remains a 'theory', at least to the general public, suggests that surveys for new data need to be taken to a new level.

## Letter to the editor

One of the most comprehensive and most important contemporary surveys has been the recently completed Census of Marine Life (Census; http://www.coml.org). This ten-year initiative involved 2,700 scientists from more than 80 countries and cost in excess of US$650 million. The Census was driven by a fundamental hypothesis, that 'there exist fundamental gaps in our knowledge and understanding of the biology of the oceans and the subsequent functioning of this system'. It has often been said that the absence of knowledge should be enough justification for exploration. The fact that the Census identified more than 6000 potentially new species and resulted in more than 2600 scientific publications validates the hypothesis. The Census community observed a potential gap in knowledge about biodiversity on our planet and wanted to fill it. Indeed this was driven not just by the scientific community, but also by public pressure for discovery. People are very responsive to new discovery, as indicated by the media response to new species found in the Census, and the questions asked of the Census by the public, e.g. 'how much biodiversity is there?'; 'why is there so much?'; 'how did it get there?'; and how much biodiversity is enough?' [1]. What has been striking has been the reinforcement of our original theory - that these voluminous observations have generated innumerable new hypotheses. This no more true than in the microbial component of the census, the International Census of Marine Microbes (ICoMM; http://icomm.mbl.edu/), which has been one of the most comprehensive studies of microbial diversity ever accomplished.

Were Darwin or his financiers around today, they would surely be deeply interested in the possibilities of exploring microbial life. Undoubtedly they would achieve this using metagenomics. In metagenomics, we isolate DNA directly from the environment and use it to characterize the taxonomy and function of the biological community in that ecosystem. The power of this approach has been lauded by contemporary scientists such as Edward O. Wilson, who famously said "...if I could start my life over, I would work in microbial ecology" [2]. As a result of metagenomic analyses over the last 30 years, we have theorized and hypothesized that the whole microbial community acts as a network providing a vast array of ecosystem services to the macro-organisms in the ecosystem. Only now and only with metagenomics, do we have the potential to produce a critical mass of data that will enable testing of these hypotheses.

Darwin started making observations about organisms before using the gathered data to generate his theory on the origin of species via natural selection. Evolution as a theory and subsequently as a testable hypothesis was generated from his open-minded observations. Likewise large-scale observational studies have aided the development of microbial ecology; for example, the Global Ocean Survey (GOS), a marine metagenomic transect survey of the world's oceans [3], has without a doubt, been one of the most influential microbial ecology studies ever. Many studies have made use of the GOS dataset to generate hypotheses and make conclusions about the biogeographic properties and functions of ocean microbial communities. Despite this clear impact, the GOS dataset is often vilified for poor experimental design and the absence of appropriate metadata necessary for analysis of the influence of environment on microbial diversity along the transect. We would agree that the data are B.A.D. - but only insofar as they are the Best Available Data - as with Darwin's imperfect survey of animals and plants, it has significantly contributed to our understanding of, in this case, marine microbiology. GOS inspired a range of metagenomic research efforts, and the resulting explosion of metagenomic discovery voyages from the human digestive tract http://www.metahit.eu; http://nihroadmap.nih.gov/hmp/ to the soil http://www.terragenome.org. Some new studies have undoubtedly used the observations made by the GOS to derive hypotheses; for example, TARA Oceans http://oceans.taraexpeditions.org/ uses a similar experimental approach to GOS, but with statistical design and contextual metadata.

The research community needs to carefully balance groundbreaking observational studies, however imperfect, with carefully designed experimental approaches. For example, the Earth Microbiome Project (EMP) http://www.earthmicrobiome.org is using metagenomics to survey the largest distribution of samples ever attempted [4,5]. While driven by specific hypotheses, which will be tested by the data, this study is also fundamentally a voyage of discovery. The 200,000 planned environmental samples sequenced for taxonomic and functional analysis, will undoubtedly generate hypotheses that are currently inconceivable. As with all data discovery, the way in which the data are analyzed and presented to the community will impact how they are used. Hence the EMP will re-assemble microbial genomes to discover new physiology, produce metabolic maps to discover functional mechanisms and explore taxonomic and protein space. The EMP is predicated on the value of voyages of discovery; and what a voyage we have before us!

The vast imbalance between what it is possible to hypothesize and test, and what is unknown means that every microbial ecologist is on an epic voyage of equal importance to that of Darwin. There are many fundamental theories about microbial life that still need to be examined, and many of these can only be explored by intelligent sampling in an unrestricted environmental surveys. Restricted analysis such as laboratory based manipulation, culturing, PCR amplification and genome experimentation are very important to the understanding of microbial adaptation. However, lab experiments are artificial and hence it will always be necessary to contextualize these results with environmental observation, and DNA extraction bias notwithstanding, metagenomics is the most unrestricted and comprehensive approach. Our ability to interpret these data is always improving [6,7] and we stand on a precipice of unprecedented discovery, such as whether the global ocean contains a homogeneous pool of microbial genes acquired by billions of years of exchange and dispersal [1]. Microbes are not the only group to benefit from these surveys; viruses exist at 10 times the abundance of microbes in virtually all ecosystems, and the only effective technique to examine the full breadth of their populations is unrestricted metagenomic survey, as no universal gene exists to allow amplicon surveys [8]. As viruses are the drivers of gene exchange - their characterization is equally necessary to answer many of the relevant questions.

Thus, funding agencies and private foundations should not reject discovery studies that aim to explore the vast frontiers of microbial life in relatively unstructured ways. This 'dark matter' must be explored, albeit intelligently. This is not a call for blind, blanket surveys, but for exploring microbial communities at a supra-ecosystem level at a time when we are realizing that we know very little about the microbial world, yet understand increasingly that microbes drive ecological processes at all scales.

Microbial ecology is rapidly evolving as a science. We need more and better surveys of every ecosystem and better standardization of ecosystem variables that can be used to relate biology to environment. The problem is vast, with more microbial life in the oceans than stars in the known universe [9], yet it is not insurmountable. There is increasing evidence, for example, for the "everything is everywhere, but the environment selects" theory being closer to the truth than anyone had previously conceived [1,10]. This is a very exciting time in global biodiversity discovery, and we must not forget that observations play a major role in science; only with effective observation can we develop testable hypotheses. It is already clear that microbial evolution, which is most of evolution, used some tricks that seem to be out of fashion among the larger organisms. So, who knows, perhaps studies like GOS, TARA and the EMP will yield the next theory of evolution, and some young Census scientist the next Darwin?

## References

[B1] O'Dor RK Fennel K Vanden Berghe E A one ocean model of biodiversity Deep-Sea Res Pt Ii 2009 56 19-20 1816 1823 10.1016/j.dsr2.2009.05.023

[B2] Wilson EO Lord of the Ants 2008 PBS: NOVA/WGBH

[B3] Rusch DB Halpern AL Sutton G Heidelberg KB Williamson S Yooseph S Wu D Eisen JA Hoffman JM Remington K The Sorcerer II Global Ocean Sampling expedition: northwest Atlantic through eastern tropical Pacific PLoS Biol 2007 5 3 e77 10.1371/journal.pbio.0050077 17355176 PMC1821060

[B4] Gilbert JA Meyer F Antonopoulos D Balaji P Brown CT Desai N Eisen JA Evers D Field D Feng W Meeting report: the terabase metagenomics workshop and the vision of an Earth microbiome project Stand Genomic Sci 2010 3 3 243 248 10.4056/sigs.1433550 21304727 PMC3035311

[B5] Gilbert JA Meyer F Jansson J Gordon J Pace N Tiedje J Ley R Fierer N Field D Kyrpides N The Earth Microbiome Project: Meeting report of the "1 EMP meeting on sample selection and acquisition" at Argonne National Laboratory October 6 2010 Stand Genomic Sci 2010 3 3 249 253 10.4056/aigs.1443528 21304728 PMC3035312

[B6] Gilbert JA Meyer F Bailey MJ The future of microbial metagenomics (or is ignorance bliss?) Isme J 2011 5 5 777 779 10.1038/ismej.2010.178 21107444 PMC3105764

[B7] Gilbert JA Dupont CL Microbial metagenomics: beyond the genome Ann Rev Mar Sci 2011 3 347 371 10.1146/annurev-marine-120709-142811 21329209

[B8] Edwards RA Rohwer F Viral metagenomics Nat Rev Microbiol 2005 3 6 504 510 10.1038/nrmicro1163 15886693

[B9] Gilbert JA Beyond the Infinite - tracking bacterial gene expression Microbiology Today 2010 37 2 82 85

[B10] Whitfield J Biogeography. Is everything everywhere? Science 2005 310 5750 960 961 10.1126/science.310.5750.960 16284155

